# Fabrication of Porous Al_2_O_3_ Ceramics with Submicron-Sized Pores Using a Water-Based Gelcasting Method

**DOI:** 10.3390/ma11091784

**Published:** 2018-09-19

**Authors:** Zhihong Yang, Nan Chen, Xiaomei Qin

**Affiliations:** 1 School of Materials Science and Engineering, Nanchang University, Nanchang 330031, China; yzhnc12@163.com; 2 Department of Physics, Shanghai Normal University, Shanghai 200234, China

**Keywords:** porous ceramics, Al_2_O_3_, submicron-sized pore, gelcasting

## Abstract

The gelcasting method is usually employed to fabricate relatively dense ceramics. In this work, however, porous Al_2_O_3_ ceramics with submicron-sized pores were fabricated using the water-based gelcasting method by keeping the Al_2_O_3_ content at low levels. By controlling the water content in the ceramic slurries and the sintering temperature of the green samples, the volume fractions and the size characteristics of the pores in the porous Al_2_O_3_ can be readily obtained. For the porous Al_2_O_3_ ceramics prepared with 30 vol.% Al_2_O_3_ content in the slurries, their open porosities were from 38.3% to 47.2%, while their median pore sizes varied from 299.8 nm to 371.9 nm. When there was more Al_2_O_3_ content in the slurries (40 vol.% Al_2_O_3_), the porous Al_2_O_3_ ceramics had open porosities from 37.0% to 46.5%, and median pore sizes from 355.4 nm to 363.1 nm. It was found that a higher sintering temperature and Al_2_O_3_ content in the slurries increased the mechanical strength of the porous Al_2_O_3_ ceramics.

## 1. Introduction

Porous materials (including ceramic, metallic and polymeric types) are being developed and employed in different fields [1,2,3,4,5,6,7,8,9,10,11,12,13,14]. Among these types of porous materials, many researchers are investigating porous ceramics [1,2,3,4,5,6,7,8,9]. To date, many methods have been developed to prepare porous ceramics. Although the partial sintering of green bodies can result in porous ceramics, their properties are not usually desirable. In general, there are three types of processing techniques [15] that have been employed to fabricate porous ceramics: replica, sacrificial template, and direct foaming.

One of the most important methods for the replica technique is the freeze-casting method [16], in which ice crystals grow in a ceramic slurry to occupy spaces inside the ceramic body. During the freeze-drying process, these ice crystals are directly vaporized by vacuum sublimation and leave pores inside the ceramic green body. The freeze-casting technique is widely used to fabricate different types of porous ceramics [17,18,19,20,21,22,23,24,25,26,27]. However, the freeze-casting process, especially the freeze-drying stage, typically takes quite a long time and consumes much more electrical power. In addition, ice crystals usually grow into dendrites, which make the pore surfaces rough, and the porous ceramics often exhibit anisotropic properties.

Herein, a water-based gelcasting route is presented for fabricating porous Al_2_O_3_ ceramics with submicron pores, which could be used for filtration and other purposes. One of the advantages of the gelcasting method is that ceramics with complicated shapes can be readily fabricated [28,29]. In this method, high solid content in the ceramic slurries, or low water content, is usually needed to obtain relatively dense ceramics [28,29]. The purpose of this work, however, is to prepare porous ceramics, rather than dense ceramics. Hence, in this work, the Al_2_O_3_ solid content is maintained at a relatively lower level, while the water content is kept at a relatively higher level in the ceramic slurries. Instead of freezing the water in the ceramic slurries into ice crystals, as in the freeze casting method [16], it is evaporated during the drying stage, and pores are retained in the green body. This allows porous Al_2_O_3_ ceramics with submicron pores to be successfully fabricated. It is noted that fabrication of porous ceramics with submicron pores using the gelcasting method has scarcely been reported in the literature.

## 2. Experimental

### 2.1. Material Preparation

Alpha Al_2_O_3_ powders (99.9% purity, grain size about 1 μm on average, Jiyuan Brother Materials Co. Ltd., Henan, China) were used in this study. The chemicals and fabrication method can be referred to in our previous work [30]. For the fabrication of porous ceramics in the present work, the Al_2_O_3_ content in the slurries was kept relatively low, at about 30–40 vol.%. In our previous work [30], however, the Al_2_O_3_ content in the slurries was about 55 vol.%, which is much higher than in the present work.

**Figure 1:**
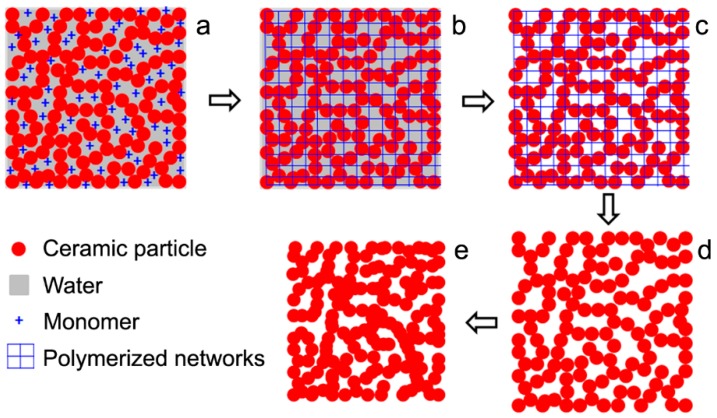
Fabrication steps of porous Al_2_O_3_ ceramics by gelcasting, (**a**) Al_2_O_3_ suspension; (**b**) polymerization of monomers; (**c**) drying; (**d**) burning out of polymers; and (**e**) sintering.

Figure 1 illustrates the processing steps for fabricating porous Al_2_O_3_ ceramics in this work. Ball-milled Al_2_O_3_ suspensions were poured into a metal mold (Figure 1a), and monomers were then polymerized to form crosslinked networks (Figure 1b) at 60 °C, for about 15 min. Then, the wet green bodies were dried at 70–110 °C and pores were retained (Figure 1c). The polymers within the dried green bodies were burnt out at 600 °C in air for 2 h. This process is called “degreasing” (Figure 1d). These samples were then sintered in air at 1300 °C, 1350 °C and 1400 °C for 2 h to obtain porous Al_2_O_3_ ceramics (Figure 1e).

### 2.2. Material Characterization

The Archimedes method was used to measure the bulk densities of the porous Al_2_O_3_ ceramics, and their flexural strength was measured with an electronic universal testing machine (Sans Materials Testing Co. Ltd., Shanghai, China) under a three-point bending setup with a span length of 30 mm and a crosshead speed of 0.5 mm/min. The size of the sample was 3 mm × 4 mm × 36 mm. For the compressive strength test, the sample size was 5 mm in diameter and 10 mm in height, and it was measured with the same instrument and the same crosshead speed. The porosities and pore sizes of the Al_2_O_3_ porous ceramics were measured using the mercury porosimetry analysis method (AutoPore IV 9500, Micromeritics, Norcross, GA, USA).

A field emission scanning electron microscope (FESEM, Hitachi S4800, Tokyo, Japan) was used to investigate the microstructural characteristics of the porous Al_2_O_3_ ceramics. The Al_2_O_3_ particle size was analyzed using an image analysis software system (ImageJ, National Institutes of Health, Bethesda, MD, USA).

## 3. Results and Discussion

### 3.1. Microstructural Characteristics

**Figure 2:**
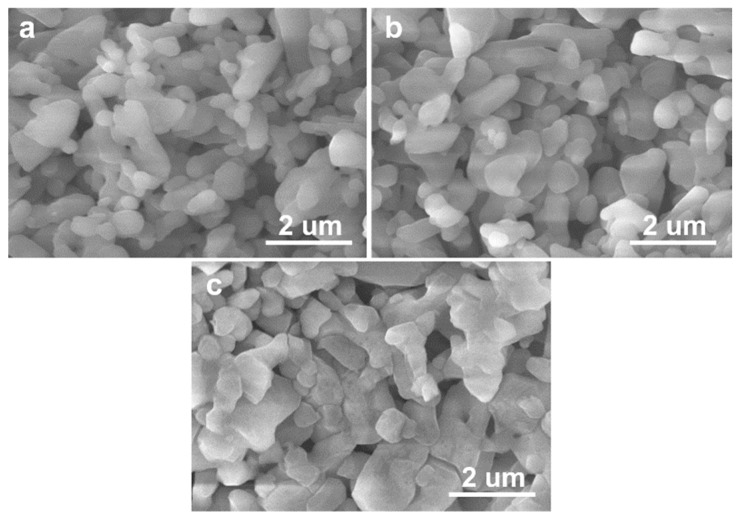
FESEM images of porous Al_2_O_3_ ceramics sintered at 1300 °C (**a**); 1350 °C (**b**) and 1400 °C (**c**), with the Al_2_O_3_ content at 30 vol.% in the ceramic slurries.

**Figure 3:**
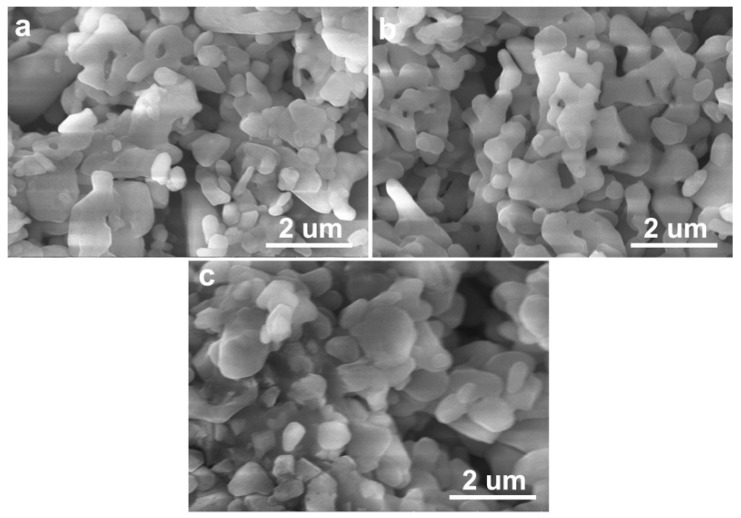
FESEM images of porous Al_2_O_3_ ceramics sintered at 1300 °C (**a**); 1350 °C (**b**) and 1400 °C (**c**), with the Al_2_O_3_ content at 40 vol.% in the ceramic slurries.

**Figure 4:**
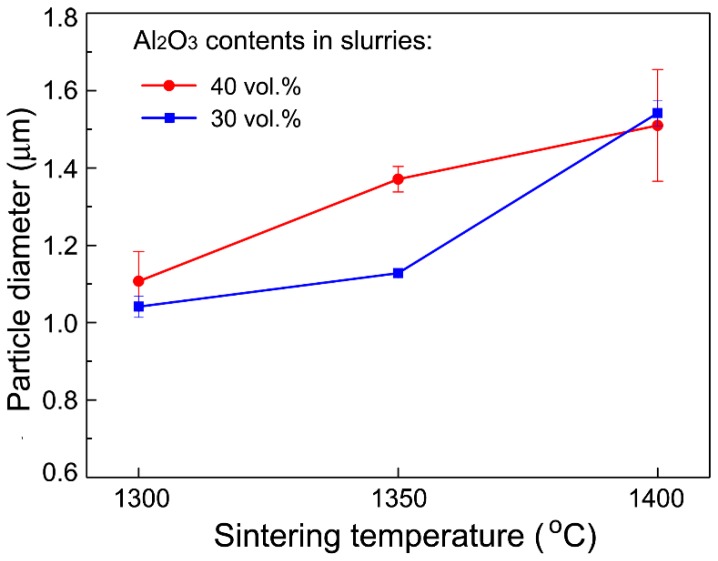
Variation of the particle diameter of porous Al_2_O_3_ ceramics with sintering temperature.

**Figure 5:**
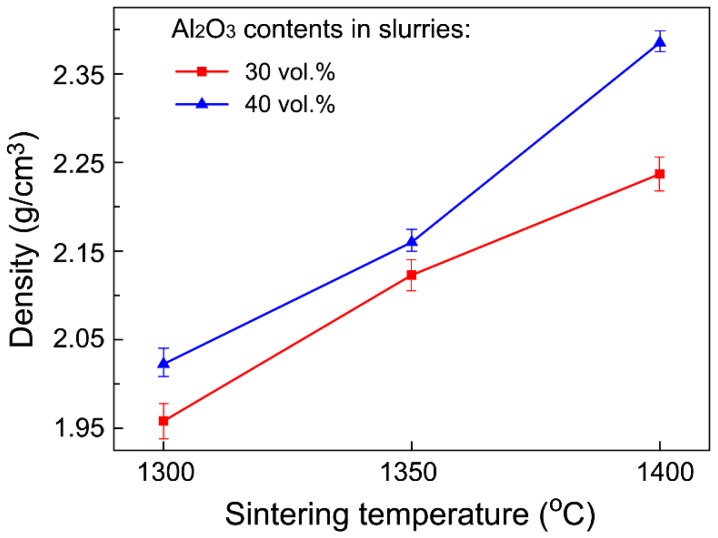
Variation of density of porous Al_2_O_3_ ceramics with sintering temperature.

Figure 2 and Figure 3 show the microstructural morphologies of the porous Al_2_O_3_ ceramics, which were prepared with the Al_2_O_3_ content in the ceramic slurries with 30 vol.% (Figure 2) and 40 vol.% (Figure 3), respectively. The pore structures can be readily seen in Figure 2 and Figure 3, and the Al_2_O_3_ particles can also be clearly identified. As shown in Figure 4 and Figure 5, the Al_2_O_3_ particle size and the density of the porous Al_2_O_3_ ceramics steadily increased with the sintering temperature. This is generally expected for ceramics [31]. For the porous Al_2_O_3_ ceramics prepared with an Al_2_O_3_ content of 30 vol.% in the slurries, the particle size and density increased from about 1.03 μm and 1.96 g/cm^3^ for sintering at 1300 °C, to 1.52 μm and 2.24 g/cm^3^ for sintering at 1400 °C, respectively. For the porous Al_2_O_3_ ceramics prepared with Al_2_O_3_ content at 40 vol.% in the slurries, the particle size and the density increased from about 1.10 μm and 2.02 g/cm^3^ for sintering at 1300 °C, to 1.49 μm and 2.38 g/cm^3^ for sintering at 1400 °C, respectively.

**Figure 6:**
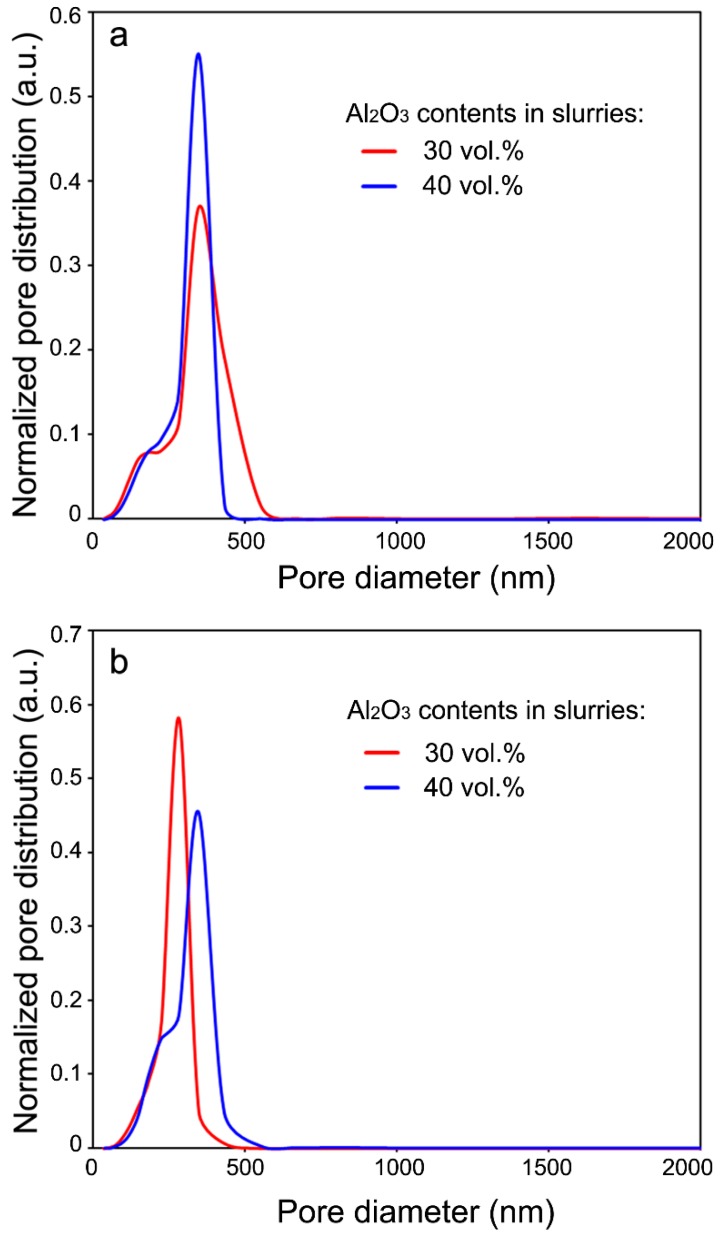
Normalized pore size distribution of porous Al_2_O_3_ ceramics for the sintering temperatures of 1300 °C (**a**) and 1400 °C (**b**).

Table 1 and Table 2 list the porosities and median pore diameters of the porous Al_2_O_3_ ceramics prepared with the Al_2_O_3_ contents in the ceramic slurries at 30 vol.% (Table 1) and 40 vol.% (Table 2), respectively. Figure 6 shows the pore size distribution functions of the porous Al_2_O_3_ ceramics sintered at 1300 °C (Figure 6a) and 1400 °C (Figure 6b). It can be seen from Table 1 and Table 2 that the porosity decreased with the sintering temperature. The closed porosities of the porous Al_2_O_3_ ceramics prepared with 40 vol.% Al_2_O_3_ content in the slurries were generally smaller than the samples prepared with 30 vol.% Al_2_O_3_ content in the slurries. In both of the two series of porous Al_2_O_3_ ceramics, the closed porosities were much smaller than the open porosities. This suggests that most of the pores in these samples were open pores [27]. This will be beneficial for filtration applications [1]. For the porous Al_2_O_3_ ceramics prepared with 30 vol.% Al_2_O_3_ content in the slurries, the median pore diameter decreased quickly from about 371.9 nm for sintering at 1300 °C, to about 299.8 nm for sintering at 1400 °C (Table 1). For the porous Al_2_O_3_ ceramics prepared with 40 vol.% Al_2_O_3_ content in the slurries, however, the pore diameter only slightly decreased (Table 2). The median pore diameter decreased from about 363.1 nm for sintering at 1300 °C, to about 355.4 nm for sintering at 1400 °C (Table 2). In fact, these results are in good agreement with the microstructural morphologies shown in Figure 2c and Figure 3c. It can be noted that the pore size in Figure 3c of the 1400 °C-sintered porous Al_2_O_3_ ceramics prepared with 40 vol.% Al_2_O_3_ content in the slurries was larger than that in Figure 2c of the 1400 °C-sintered porous Al_2_O_3_ ceramics prepared with 30 vol.% Al_2_O_3_ content in the slurries.

### 3.2. Mechanical Properties

**Figure 7:**
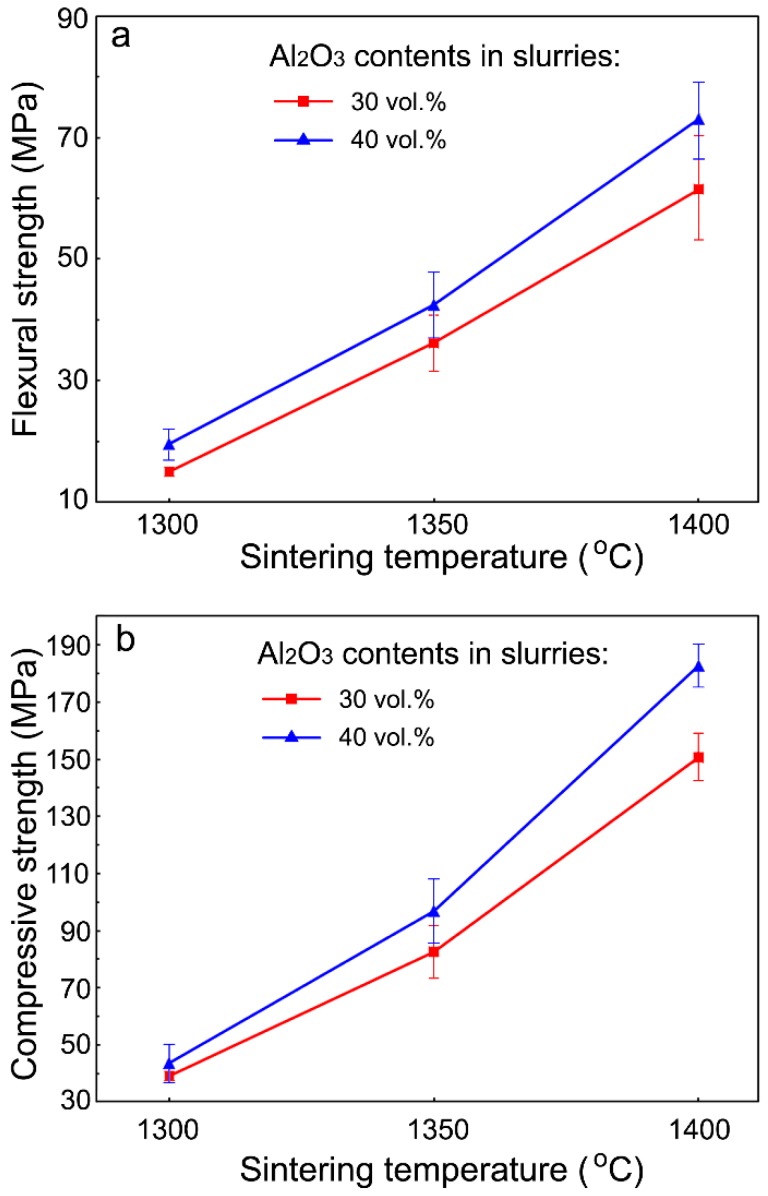
Variation of the flexural strength (**a**) and compressive strength (**b**) of porous Al_2_O_3_ ceramics with sintering temperature.

The flexural and compressive strength of the porous Al_2_O_3_ ceramics are shown in Figure 7. Figure 7a shows the dependence of the flexural strength of the porous Al_2_O_3_ ceramics on the sintering temperature. The flexural strength increased with the increasing sintering temperature. For the porous Al_2_O_3_ ceramics prepared with Al_2_O_3_ content at 30 vol.% in the slurries, their flexural strength increased from 15.0 MPa when sintered at 1300 °C, to 36.2 MPa and 61.5 MPa when sintered at 1350 °C and 1400 °C, respectively. For the porous Al_2_O_3_ ceramics prepared with Al_2_O_3_ content at 40 vol.% in the slurries, their flexural strength increased from 19.6 MPa when sintered at 1300 °C, to 42.5 MPa and 73.1 MPa when sintered at 1350 °C and 1400 °C, respectively. Compared with our previous work on gelcasted Al_2_O_3_ ceramics [30], in which 55 vol.% Al_2_O_3_ content was used in the slurries, the porous Al_2_O_3_ ceramics of the present work had smaller flexural strength.

As shown in Figure 7a, in general, the porous Al_2_O_3_ ceramics prepared with 40 vol.% Al_2_O_3_ content in the slurries exhibited higher flexural strength than those prepared with 30 vol.% Al_2_O_3_ content in the slurries for all three sintering temperatures. Furthermore, the difference in their flexural strength became larger at the higher sintering temperature of 1400 °C (Figure 7a). This can be attributed to the larger total porosity of the porous Al_2_O_3_ ceramics prepared with 30 vol.% Al_2_O_3_ content in the slurries than those with 40 vol.% Al_2_O_3_ content in the slurries (Table 1 and Table 2).

The compressive strength of the porous Al_2_O_3_ ceramics increased with the increasing sintering temperature (Figure 7b). This variation in behavior is similar to the flexural strength as shown in Figure 7a. For the porous Al_2_O_3_ ceramics prepared with Al_2_O_3_ content at 30 vol.% in the slurries, their compressive strength increased from 39.1 MPa when sintered at 1300 °C, to 82.6 MPa and 150.6 MPa when sintered at 1350 °C and 1400 °C, respectively. For the porous Al_2_O_3_ ceramics prepared with Al_2_O_3_ content at 40 vol.% in the slurries, their compressive strength increased from 43.6 MPa when sintered at 1300 °C, to 96.9 MPa and 182.8 MPa when sintered at 1350 °C and 1400 °C, respectively. Therefore, these porous Al_2_O_3_ ceramics are mechanically strong enough for practical applications. The dependence of their compressive strength on the sintering temperature (Figure 7b) is in agreement with the results of the Al_2_O_3_/mullite composite porous ceramics reported by others [32]. However, the compressive strength of the porous Al_2_O_3_ ceramics of this work was consistently higher than the Al_2_O_3_/mullite composite porous ceramics [32] and the porous Al_2_O_3_ ceramics prepared using carbon black as a pore former [33].

## 4. Conclusions

Porous Al_2_O_3_ ceramics with submicron pores were fabricated using the water-based gelcasting method. The open porosities and median pore sizes of the porous Al_2_O_3_ ceramics with 30 vol.% Al_2_O_3_ content in the slurries were 47.2% and 371.9 nm when sintered at 1300 °C, 42.5% and 330.6 nm when sintered at 1350 °C, and 38.3% and 299.8 nm when sintered at 1400 °C. The open porosities and median pore sizes of the porous Al_2_O_3_ ceramics with 40 vol.% Al_2_O_3_ content in the slurries were 46.5% and 363.1 nm when sintered at 1300 °C, 41.7% and 358.5 nm when sintered at 1350 °C, and 37.0% and 355.4 nm when sintered at 1400 °C. The porous Al_2_O_3_ ceramics exhibited high mechanical strength, which increased with both increasing sintering temperature and increasing Al_2_O_3_ content in the slurries.

## Figures and Tables

**Table 1:** Porosities and median pore diameters of porous Al_2_O_3_ ceramics (30 vol.% Al_2_O_3_ in the slurries).

Sintering Temperature (°C)	Open Porosity (%)	Closed Porosity (%)	Median Pore Diameter (nm)
1300	47.2	1.6	371.9
1350	42.5	2.2	330.6
1400	38.3	3.2	299.8

**Table 2:** Porosities and median pore diameters of porous Al_2_O_3_ ceramics (40 vol.% Al_2_O_3_ in the slurries).

Sintering Temperature (°C)	Open Porosity (%)	Closed Porosity (%)	Median Pore Diameter (nm)
1300	46.5	0.8	363.1
1350	41.7	1.9	358.5
1400	37.0	0.9	355.4
